# How to reach optimal estimates of confidence intervals in microscopic counting of phytoplankton?

**DOI:** 10.1093/plankt/fbab062

**Published:** 2021-10-04

**Authors:** Kalevi Salonen, Pauliina Salmi, Jorma Keskitalo

**Affiliations:** Lammi Biological Station, University of Helsinki, Pääjärventie 320, FI-16900 Lammi, Finland; Faculty of Information Technology, University of Jyväskylä, PO Box 35, Jyväskylä FI-40014, Finland; Lammi Biological Station, University of Helsinki, Pääjärventie 320, FI-16900 Lammi, Finland

**Keywords:** confidence intervals, dynamic counting, microscopy, phytoplankton

## Abstract

Present practices in the microscopic counting of phytoplankton to estimate the reliability of results rely on the assumption of a random distribution of taxa in sample preparations. In contrast to that and in agreement with the literature, we show that aggregated distribution is common and can lead to over-optimistic confidence intervals, if estimated according to the shortcut procedure of Lund *et al.* based on the number of counted cells. We found a good linear correlation between the distribution independent confidence intervals for medians and those for parametric statistics so that 95% confidence intervals can be approximated by using a correction factor of 1.4. Instead, the recommendation to estimate confidence intervals from the total number of counted cells according to Lund *et al.* should be categorically rejected. We further propose the adoption of real-time confidence intervals during microscopic counting as the criterion to define how long counting should be continued. Then each sample can be counted in its individual way to reach the necessary reliability independent of highly different samples. Such a dynamic counting strategy would be the most significant development in the quality control of phytoplankton counting since the early pioneers established the present counting practices in the late 1950s.

## INTRODUCTION

Microscopic counting of phytoplankton is one of the basic determinations used in the assessment of the ecological status of aquatic environments. Generally, a settling chamber technique (Utermöhl, 1958) is applied according to standardized approaches (e.g. EN 15204, 2006). Significant effort has been made to ascertain the comparability of the results by various measures of quality assurance (e.g. Rott *et al.*, 2007). Although there is a need for universal counting approaches, it is illusory to believe that all problems (Rott, 1981; Thackeray *et al.*, 2013) can be solved by adherence to any single standard. Despite their merits, standardized approaches may even have engendered a false feeling of adequate quality control of counting results. For example, quality assurance is only halfway towards good phytoplankton results and does not guarantee their comparability (Duarte *et al.*, 1990). Quality assurance increases the likelihood of achieving good results, but only quality control can document whether they are realized.

Many factors like sampling, preservation, subsampling, settling and counting contribute to the total variation of the results but have surprisingly rarely been taken into account. Chemical, biological and physical (e.g. Blukacz *et al.*, 2009) factors can lead to patchy vertical and temporal distributions in water bodies so that sampling generally contributes to the bulk of the variation in the results. Similarly, the variation across lakes has been largely neglected. Microscopic counting is typically the next most important source of variation (Kirchman *et al.*, 1982). In particular, the rarity of confidence interval estimations weakens the usefulness of phytoplankton count data.

Lund *et al.* (1958) emphasized the crucial importance of the confidence intervals of microscopic counts. At a time when no personal computers or even calculators were available, they suggested a shortcut procedure to estimate confidence intervals based on the assumption that the spatial distribution of specimens on a settling chamber is random (Poisson distributed). However, various factors can lead to an aggregated (variance > mean) distribution of phytoplankton on the settling chamber. Variation in temperature during settling is one of the most critical aspects, because it can produce density gradient currents in the settling chamber so that lateral or radial differences in cell density may develop (Sandgren and Robinson, 1984).

Count data of ecological studies regularly violate the equidispersion constraint imposed by the Poisson distribution (Lynch *et al.*, 2014). Accordingly, empirical observations (Nauwerck, 1963; Rott, 1981; Sandgren and Robinson, 1984) and simulations (Edgar and Laird, 1993) have demonstrated a common lack of spatial randomness of settled phytoplankton. Nevertheless, when confidence intervals are shown in the literature, they are generally (Edgar and Laird, 1993) derived from the total number of counts according to the shortcut procedure of Lund *et al.* (1958). Even more often, no confidence intervals are given at all (Edgar and Laird, 1993). Thus, despite convincing evidence that the distribution of phytoplankton cells on the settling chamber is often aggregated, it has not been addressed in microscopic counting since the early pioneers established the present counting practices more than 60 years ago.

The purpose of this study was to have a deeper insight into the distribution of phytoplankton cells on counting chambers to find an objective basis to reach reliable confidence limits of microscopic counts with minimum work effort. We hypothesized that irrespective of the phytoplankton distribution on the settling chamber, a satisfactory approach can be found to calculate the confidence intervals of the results to advance the interpretation of the results.

## MATERIALS AND METHODS

### Sample preparation

Lake water samples were preserved with Lugol’s iodine (0.5–1 mL in 100 mL of water). Before settling (Utermöhl, 1958), samples were acclimated to the prevailing laboratory temperature for 1 day and then the bottles were gently mixed by turning them up and down for ca 2 min. The 25 mm diameter settling chambers were kept on a table protected from direct sunlight and airflow for at least 8 h (10 mL chambers) or 24 h (25 mL and 50 mL chambers).

### Counting of samples

Settled phytoplankton samples were counted by two researchers. One counted winter samples from one lake and the other counted summer samples from different lakes. Both used an inverted light microscope with phase-contrast optics (Wild M40, Switzerland and Olympus IX50, Japan) with 300×, 400× or 600× magnification depending on the size and abundance of taxa. Unicellular phytoplankton species were counted from replicate microscopic fields selected pseudorandomly, whereby fields for counting were located by blindly moving the microscope stage to a new position. In practice, any area close to the margin of the settling chamber was avoided. A proprietary computer program (open version in preparation) was used to register the results of individual fields of view.

The results of this study were taken from routine countings where phytoplankton biomass was the primary object. We only used counts of solitary taxa with the total number of counted specimens per sample >50, and the number of counted fields at least 10 (up to 30, mean 14). Although the number of cells counted for individual species often remained rather small, our results probably represent well the reality of routine countings.

### Statistical analyses

The relative 95% parametric confidence intervals (PCIs) of the results as a percentage of the mean were calculated according to:

(1) \begin{equation*} \mathrm{PCI}=200\ast \frac{{\mathrm{t}}_{0.025\ast}\sqrt{s^2}}{\rm{mean}\ast\sqrt{n}} \end{equation*}

where t_0.025_ is the 97.5% percentile of the *t*-distribution with n − 1 degrees of freedom, *s*^2^ is sample variance and n is the number of replicates. Because variance and mean are equal in the Poisson distribution, Lund *et al.* (1958) substituted the sample variance of equation (1) by the sample mean and derived a shortcut equation to calculate a confidence interval (LCI) as a percentage of the mean:

(2) \begin{equation*} \mathrm{LCI}=2\ast \frac{200}{\sqrt{\mathrm{N}}} \end{equation*}

where *N* = total number of counted specimens and the *t*-value was set equal to 2, which is a practical approximation, if the number of counted fields is >30.

We also calculated confidence intervals for the median and computationally simple BP median (Bonett and Price 2002), which are distribution independent and hence robust. Lower and upper ranked values (RV) were calculated according to Campbell and Gardner (2000) to approximate the confidence intervals for medians:

(3) \begin{equation*} \mathrm{RV}=0.5\mathrm{n}\pm 1.96\ast \sqrt{\left(\mathrm{n}\ast 0.5\ast \left(1-0.5\right)\right)} \end{equation*}

where *n* = number of replicates.

IBM SPSS Statistics Version 20 (IBM, Armonk, NY, USA) was applied for statistical analyses. The Student’s t-test was used to test the differences between two independent groups. Before all tests, Levene’s test was used to test the equality of variances and Shapiro–Wilk’s test was used to test normality. If the assumptions of the t-test were not fulfilled, the Mann–Whitney U test was chosen.

## RESULTS

The results of phytoplankton (11 taxa) counted on settling chambers (*n* = 113) showed a rather high range of the coefficient of variation (mean CV 43%, range 16%–90%) between replicate microscope fields. The median counts were on average 4.3% lower (CV 219%) than the mean counts (range 3–161) but the difference was not significant (Mann–Whitney U test, *P* = 0.52).

**Fig. 1:**
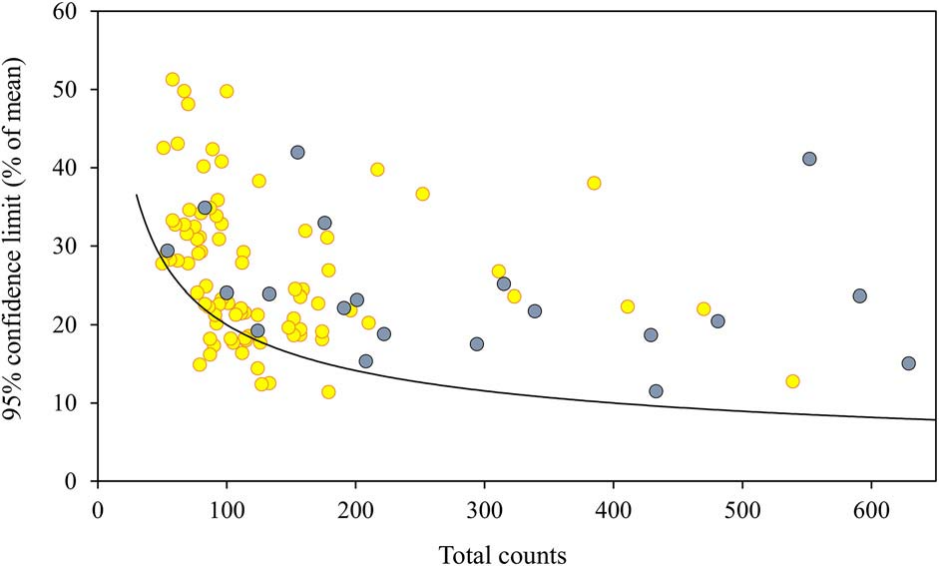
Comparison between Lund based (LCI/2, curve) and parametric statistics based (PCI/2, dots) 95% confidence limits in relation to the number of counted cells. Black dots—*Stephanodiscus* samples.

**Fig. 2:**
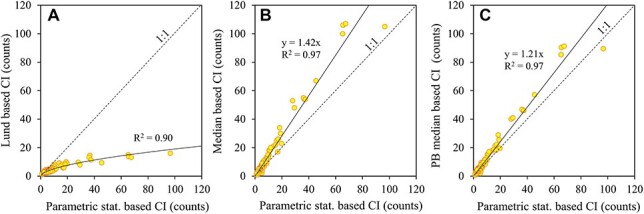
Comparison of Lund *et al.* (1958) (A), median (B) and BP median (C) based 95% confidence intervals (CI) of phytoplankton counts with parametric statistics based confidence intervals. The 1:1 line shows where the confidence intervals of both approaches yield equal results. Each point represents an individual sample.

Different approaches to calculate confidence limits of averages produced markedly different results. The relative confidence limits of means calculated according to the parametric statistics (Fig. 1) were often several times wider than those calculated according to the shortcut procedure of Lund *et al.* (1958) independently of the total number of counted cells. According to the Lund approach confidence limits, roughly 10% of the mean, were obtained when about 400 cells were counted, but in reality the values were often 2–3 times wider. A comparison between the results of these two methods shows that the discrepancy increased non-linearly with increasing confidence interval (Fig. 2A). The respective relationship for the median was linear and departed from the 1:1 reference line with a 40% steeper slope (Fig. 2B). BP median similarly showed wider confidence intervals compared to the parametric statistics but with only ~20% difference (Fig. 2C).

**Fig. 3:**
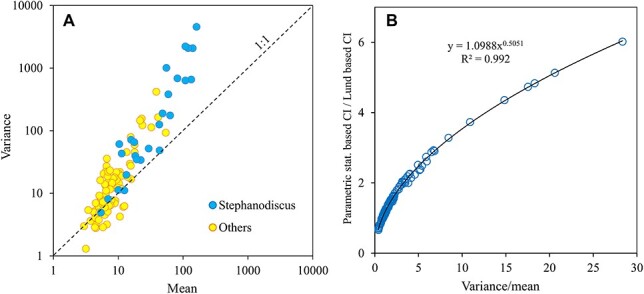
Relationship between variance and mean of cell counts of phytoplankton (A) and dependence of parametric/Lund statistics based estimates of confidence intervals from variance-to-mean ratio (B).

The variance-to-mean ratio of the counts was often >1, indicating an aggregated distribution of the cells (Fig. 3A). The highest aggregation was due to a small (diameter ca 5 μm) *Stephanodiscus* cf. *parvus* diatom, which was very abundant in an ice-covered lake. However, the regressions between the logarithms of variance and mean for *Stephanodiscus* and the other taxa were not far from each other (<11% difference in regression coefficients). The variance-to-mean ratio of microscopic counts of settled phytoplankton taxa almost perfectly explained the difference between the confidence intervals based on parametric statistics and those based on the shortcut procedure of Lund (Fig. 3B). In contrast, the respective comparison with median-based statistics showed no significant relationship (*R*^2^ < 0.01). The upper confidence limits for mean, median and BP median were close to each other, but the lower ones for median and BP median were on average 29 *±* 3% and 16 *±* 4% (with 95% confidence limits), respectively, wider than those of parametric statistics.

## DISCUSSION

When the results of individual microscopic fields have seldom been used to calculate the confidence limits, the only indication of the reliability of the results has often been through intercalibrations, where replicate subsamples have been counted by the same or several persons. Vuorio *et al.* (2007) reported CVs for 20 fields of two samples counted by the same person. In both cases of *Rhodomonas* counting, CV was 13% and in those of *Fragilaria* counts 14% and 19%. Respective results of counts through the diameter of the settling chamber were 10% and 15% as well as 5% and 23%. Although the number of counted cells was not reported, these results seem to represent the lowest range of variation in our study.

When our results are compared with published intercalibration results where parallel subsamples have been counted by several microscopists (Willén 1976), the CVs of our results were on average almost two times higher (23% vs. 45%; *n* = 90 vs. 113) in samples with at least 50 counted cells. However, Hobro and Willén (1977) who reported results of subsamples counted in three laboratories (in each case 10 parallel subsamples) found higher average CVs of 45% (range10%–57%), 51% (range 14%–90%) and 12% (range 4%–13%), respectively, which rather closely corresponds our results. Because the variation of the counts between counting fields is inherently higher than the variation between the mean counts of replicate fields, our results seem to be in line with the intercalibrations. As our samples originated from many different lakes with different taxa, our counting results are likely representative of routine phytoplankton counting in general.

Variance-to-mean ratios generally higher than unity verified that the aggregated distribution of phytoplankton on counting chambers is the rule rather than the exception (Fig. 3A). The observed extremely high variance-to-mean ratios of *Stephanodiscus* diatoms may be explained by delicate polysaccharide fibrils of diatoms (Svetličić *et al.*, 2013), which in our samples were faintly visible under a phase-contrast microscope. The preservation of the samples may have facilitated the adherence of cells together by the fibrils.

In agreement with the simulations of Edgar and Laird (1993) and earlier field observations, in our samples, the shortcut approach of Lund *et al.* (1958) often yielded unrealistically low confidence intervals. At a time when no calculators were available, the shortcut approach was no doubt useful to indicate how much work should be allocated for counting and the same is still true. However, in the computer age, the Lund approach should be consigned to history as an indicator of the confidence intervals of the results. Instead, counting programs capable of providing reliable confidence limits in real-time should be preferred. Then it is possible to have a good idea about the reliability of the results in so early a phase that counting effort can be allocated to reach the desired level of certainty or to focus on the most critical taxa of the sample concerning the uncertainty of the results. It is noticeable that dynamic counting does not increase work but can make it more efficient. It is also more motivating, as results can be seen immediately and their processing can start already during counting. There are a few phytoplankton counting programs in the market, but their scope is limited to serve as a simple calculator rather than to support the quality of the results. Thus, at the moment the limiting factor is the availability of suitable programs.

In the simulations of Edgar and Laird (1993), the range of the variance-to-mean ratio was modest (0.6–2.2) and parametric statistics based confidence limits produced a reasonable agreement with an expected error rate of 0.05. In our results, the range of the variance-to-mean ratio was an order of magnitude higher (0.3–28.2) and the results revealed on average much wider confidence intervals for the median compared with parametric statistics based ones. Thus, straightforward use of parametric statistics can lead to a too optimistic interpretation of the results. The confidence limits for the median are robust but asymmetrical to the median value and computationally more complicated (BP median is more convenient). Fortunately, the linear relationship between the CIs of the mean and the median allows the use of a simple empirical correction factor of 1.4 to probably reach the most realistic estimates. In comparison with the confidence limits based on the Lund approach, the correction dramatically improves the evaluation of the results of samples with high aggregation of cells. Consequently, our findings verified the hypothesis that distribution independent confidence intervals for microscopic counts can be derived from parametric statistics.

### Implications for phytoplankton counting

Few words may be necessary to comment on what is the most representative average value of microscopic counts. Because the median minimizes the effect of a significant number of outliers in the data, it is sometimes favoured as a robust average value. In our samples, with spuriously high counts in microscopic fields as well as in samples with empty fields, the median was typically smaller than the mean, but the difference was in practice negligible. Similarly, Rott (1981) has found that the elimination of outliers does not markedly affect phytoplankton counting results. Further, if we exclude clear mistakes, such as typing errors, “outliers” in phytoplankton counts are generally not real errors and hence their omission biases the mean results. Consequently, outliers should not be rejected without strong a priori reason. On the other hand, because the calculation of the median does not increase work, we suggest that in phytoplankton counting both the arithmetic mean and median values should be provided to assist the interpretation of results. Because of the higher stability of the median, such a practice might sometimes strengthen the conclusions.

Despite decades of extensive availability of computers, their power has generally only been used for the same routine calculations as 60 years ago. As emphasized by the high variance-to-mean ratio of *Stephanodiscus* diatoms in this study, not too much weight should be given to the results of occasional intercalibrations, which have no general validity for individual phytoplankton samples. Instead, it should be mandatory to have an idea about the quality of the results based on the statistics of observed results rather than on any assumptions. In this way, the results could be most realistically interpreted. Here it should also be noted that the counting of samples is only one source of variation.

In the present European phytoplankton counting standard (EN 15204, 2006), confidence intervals calculated from individual counts in microscopic fields are recommended in parallel with the shortcut approach of Lund *et al.* (1958). However, in practice the recommendation remains largely unrealized, because the necessary calculations are tedious. Thus, there is an urgent need for phytoplankton counting programs, such as that used in our study, which provide confidence intervals in real-time.

Phytoplankton counting instructions often advise verification that specimens are randomly distributed and suggest the preparation of a new sample, if this is not fulfilled. However, visual inspection of distribution is subjective and is of value only for the most striking deviations from randomness in quite dense phytoplankton samples. Second, a strictly stable settling temperature to exclude convective water circulation and its effect on the distribution of cells in the settling chamber may be difficult to achieve particularly in summer, when there is often no compensation for diurnally fluctuating indoor temperature. Hence, convection can affect the distribution of cells (Berthold and Resagk 2012*)*. Third, as demonstrated by the *Stephanodiscus* diatom, sometimes the reason for the aggregated distribution is an inherent feature of the sample rather than the settling conditions. Maybe the only efficient way to minimize an aggregated distribution of cells is a careful acclimation of samples to the temperature of the settling environment and to prevent vibrations as well as temperature fluctuation.

In addition to the estimation of the uncertainty of the results, the distribution of phytoplankton in a settling chamber has important ramifications for how samples should be counted. The finding of Sandgren and Robinson (1984) that cell abundances near the margin of the settling chamber can be higher than around the centre poses a significant challenge in microscopic counting. Commonly used so-called pseudorandom selection of fields for counting tends to lead to the under-representation of the marginal zone of the settling chamber and consequent systematic underestimation of cell count. Four alternative approaches might be used: stratified counting of the central and marginal areas of the settling chamber, diameter counting, true random selection of fields and stripe counting. Stratified counting partly compensates for a radially uneven distribution of cells, but because the proportion of the chamber area non-linearly increases with the distance from the centre, the problem is only alleviated, not controlled. For the same reason, the widely applied diameter counting cannot be recommended, although it has often been erroneously assumed to compensate for differences in the distribution of phytoplankton over the settling chamber. Counting of truly random fields is theoretically an ideal solution, but without a computer-run motorized stage it remains by far too impractical. Instead, simple counting of evenly distributed stripes, which compensates for both laterally and radially non-random distributions of phytoplankton cells, can be recommended. Interlaced replicate sets of stripes might be used to estimate confidence intervals. A new counterpart for random counting might also be provided by Archimedean spirals marked at equal distances along their lengths on the bottom glass of the settling chamber. When all fields are counted at even distances from each other through the whole spiral the counting would represent the whole chamber area in correct proportion. Compared with stripe counting, this method is more flexible to cope with a range of abundances, because the number of points to be counted in the spiral can be varied.

## CONCLUSIONS

Present phytoplankton counting practices cannot optimally cope with various qualities of samples or different goals of counting. Because an aggregated distribution of specimens is common, and often technically unavoidable, the deterioration of the quality of the results should be compensated for by a proper counting procedure. Our results suggest that confidence intervals derived from parametric statistics can provide distribution independent and reliable information on the quality of the results when corrected by the empirical factor of 1.4.

In addition to how long the counting of each sample or taxon should be continued to reach wanted confidence intervals of abundance, dynamic counting has important ramifications for the estimation of phytoplankton biomass. It provides an unprecedented possibility to allocate work effort so that the vast size and abundance range of taxa can be taken into account. When necessary, any taxon can be counted in its specific way. This is in striking contrast with the paradigm that in routine counting a fixed number of views or cells is assumed to produce the most comparable results. Because of highly different samples, it is not always the case. With an appropriate counting program, complicated calculations are automatic and there is no problem applying whatever mixture of counting strategies within one sample without increasing the work effort. We have successfully applied dynamic counting for counting bacterioplankton and phytoplankton (Salmi *et al.*, 2014) to adjust the quality of the results to the level necessary for the goal of the study during the ongoing microscopy sessions, i.e. at the only time when it is still possible.

Dynamic counting of phytoplankton allows the microscopist to decide objectively how to balance work effort between the quality of the results and available resources. Thus, it can provide a universal and objective platform suitable for any samples and targets. Future standardization no longer needs to recommend how many counts (or counted fields, etc.) are needed but rather what confidence intervals should be reached. We believe that dynamic counting will be one of the greatest advances in microscopic phytoplankton counting since the early pioneers established the present settling chamber practices. Its introduction would be quite straightforward, because the creation of appropriate computer programs is not difficult.
